# Pre-hospital critical care anaesthesiologists and traumatic brain injury-guideline adherence

**DOI:** 10.1186/1757-7241-22-S1-P7

**Published:** 2014-07-07

**Authors:** Leif Rognås, Troels Martin Hansen, Hans Kirkegaard, Else Tønnesen

**Affiliations:** 1 Department of Research and Development, Norwegian Air Ambulance Foundation, Drøbak, Norway; 2 Pre-hospital Critical Care Team, Aarhus University Hospital, Aarhus, Denmark; 3 Centre for Emergency Medicine Research, Aarhus University Hospital, Aarhus, Denmark; 4 Department of Anaesthesiology, Aarhus University Hospital Aarhus, Denmark

## Background

Guidelines recommend that brain trauma patients with a Glasgow Coma Scale score <9 should have an airway established. Also, SpO_2_ >90%, systolic blood pressure >90 mmHg and end-tidal CO_2_ between 4.5 and 5.3 kPa is advised [1]. The objectives were to investigate guideline adherence, reasons for non-adherence and the incidences of complications related to pre-hospital advanced airway management in traumatic brain injury patients.

## Materials and methods

We prospectively collected data [2] from eight anaesthesiologist-staffed pre-hospital critical care teams in the Central Denmark Region according to the Utstein-style template [3].

## Results

Among 1081 consecutive pre-hospital advanced airway management patients, we identified 54 with a traumatic brain injury and an initial Glasgow Coma Scale score <9. Guideline adherence regarding airway management was 92.6%. Reasons for non-adherence were patient’s condition, anticipated difficult airway management and short distance to the emergency department. Following rapid sequence intubation, 11.4% suffered an oxygen saturation <90%, 9.1% had a first post-rapid sequence intubation systolic blood pressure <90 mmHg and 48.9% had a first post-rapid sequence intubation systolic blood pressure <120 mmHg. The incidence of hypertension following pre-hospital rapid sequence intubation was 4.5%. The incidence of post-endotracheal intubation hyperventilation was 71.1%.

## Conclusion

The adherence to airway management guidelines was high. The incidences of post-rapid sequence intubation hypoxia and systolic blood pressure <90 compare to results reported from other physician-staffed pre-hospital services. The incidence of systolic blood pressure <120 as well as that of hyperventilation following pre-hospital endotracheal intubation in traumatic brain injury patients call for a change in our current practice.
